# Study of Ras Mutations’ Prognostic Value in Metastatic Colorectal Cancer: STORIA Analysis

**DOI:** 10.3390/cancers12071919

**Published:** 2020-07-16

**Authors:** Alessandro Ottaiano, Nicola Normanno, Sergio Facchini, Antonino Cassata, Anna Nappi, Carmela Romano, Lucrezia Silvestro, Alfonso De Stefano, Anna Maria Rachiglio, Cristin Roma, Monica R. Maiello, Stefania Scala, Paolo Delrio, Fabiana Tatangelo, Annabella Di Mauro, Gerardo Botti, Antonio Avallone, Guglielmo Nasti

**Affiliations:** 1 Department of Abdominal Oncology, SSD-Innovative Therapies for Abdominal Cancers, Istituto Nazionale Tumori di Napoli, IRCCS “G. Pascale”, Via M. Semmola, 80131 Naples, Italy; sergio.facchini.93@gmail.com (S.F.); g.nasti@istitutotumori.na.it (G.N.); 2 Cell Biology and Biotherapy Unit, Istituto Nazionale Tumori di Napoli, IRCCS “G. Pascale”, Via M. Semmola, 80131 Naples, Italy; n.normanno@istitutotumori.na.it (N.N.); anmarachiglio@yahoo.it (A.M.R.); cristin.roma@gmail.com (C.R.); monicmaiello@yahoo.it (M.R.M.); 3 Department of Abdominal Oncology, Experimental Clinical Abdominal Oncology, Istituto Nazionale Tumori di Napoli, IRCCS “G. Pascale”, Via M. Semmola, 80131 Naples, Italy; a.cassata@istitutotumori.na.it (A.C.); a.nappi@istitutotumori.na.it (A.N.); c.romano@istitutotumori.na.it (C.R.); l.silvestro@istitutotumori.na.it (L.S.); a.destefano@istitutotumori.na.it (A.D.S.); a.avallone@istitutotumori.na.it (A.A.); 4 Functional Genomics, Istituto Nazionale Tumori, IRCCS “G. Pascale”, Via M. Semmola, 80131 Naples, Italy; scalaste@gmail.com; 5 Department of Abdominal Oncology, Colorectal Surgery, Istituto Nazionale Tumori di Napoli, IRCCS “G. Pascale”, Via M. Semmola, 80131 Naples, Italy; p.delrio@istitutotumori.na.it; 6 Department of Pathology, Istituto Nazionale Tumori di Napoli, IRCCS “G. Pascale”, Via M. Semmola, 80131 Naples, Italy; f.tatangelo@istitutotumori.na.it (F.T.); annab.dimauro@gmail.com (A.D.M.); g.botti@istitutotumori.na.it (G.B.)

**Keywords:** KRAS, NRAS, prognosis, colorectal cancer, survival

## Abstract

Background: Colorectal cancer (CRC) is the second most common cause of cancer-specific death in both sexes in Western countries. KRAS mutations occur in about 50% of metastatic CRCs (mCRCs). The prognostic value of specific KRAS mutations still remains unexplored and unclear. Methods: Two hundred and forty KRAS wild-type and 206 KRAS/NRAS mutant consecutive unresectable mCRC patients with PS Eastern Cooperative Oncology Group (ECOG) 0 or 1, aged < 80 years, and with a life expectancy >3 months entered into this study. DNA was extracted from paraffin-embedded formalin-fixed tumour tissues, and it was sequenced with the Oncomine Solid Tumour DNA kit (Thermo Fisher Scientific, Waltham, MA, USA). Data were analysed using the Torrent Suite Software v5.0 (Thermo Fisher Scientific). The primary outcome was the analysis of the prognostic role of different KRAS mutations in terms of overall survival (OS). Results: There were no significant differences among the most prevalent mutations (p.G12D, p.G12V, p.G13D, p.G12A, p.G12C, and p.G12S) in terms of age (<65 vs. ≥65 years), gender (male vs. female), grading (G1/G2 vs. G3), side of primary tumour (left vs. right), pT, and pN. At the median follow-up of 25.6 months, there were 77 deaths in KRAS-mutated patients and 94 in wild-type patients. Three homogeneous prognostic groups were identified: wild-type patients (group A, median survival: 27.5 months), p.G13D/p.G12A/p.G12V/p.G12D mutants (group B, median survival: 17.3 months), and p.G12C/p.G12S mutants (group C, median survival: 5.0 months, *p* < 0.0001 according to Log Rank test). Upon multivariate analysis, metastatic involvement and p.G12C/p.G12S KRAS mutation group C (vs. other mutations) emerged as independent prognostic variables for survival. Conclusions: We show that mutant KRAS is a negative prognostic factor and that p.G12C/p.G12S variants present the worst clinical courses. This information suggests a clear difference among KRAS mutations, and it will be useful to test potentiated and/or innovative therapeutic strategies in p.G12C/p.G12S metastatic CRC patients.

## 1. Introduction

Colorectal cancer (CRC) is a challenging disease, being responsible for millions of deaths each year in Western countries, and it is the third most frequent type of cancer and the second most common cause of cancer-specific death in both sexes [1]. Despite the application of screening programs, about half of patients still present with metastatic CRC (mCRC), typically involving the lymph nodes, liver, lungs, and peritoneum [2,3]. Today, the median survival of advanced patients surpasses 24 months due to the recent introduction of new chemotherapy drugs (fluorouracil, irinotecan, and oxaliplatin) and biological drugs (bevacizumab, aflibercept, cetuximab, panitumumab, and regorafenib) [3].

Treatments’ results have been improved through (i) an increasing understanding of CRC biology and genetics and (ii) better patient selection. RAS proteins (in detail, KRAS—Kirsten RAt Sarcoma viral oncogene homolog—and NRAS—Neuroblastoma RAS viral oncogene homolog) are small GTPases (Guanosine TriPhosphatases) involved in the EGFR (Epidermal Growth Factor Receptor) pathway [4]. When activated by ligand/receptor binding, RAS protein switches on and activates, in turn, crucial kinases (i.e., phosphoinositide 3-kinase (PI3K)-AKT (serine-threonine kinase inducing AKr Thymic lymphoma), RAF (Rapidly Accelerated Fibrosarcoma), etc.) involved in stimulating a plethora of cancer-related phenomena (migration, survival, adhesion, growth, and differentiation) [5].

Many studies have demonstrated that KRAS is mutated in about 50% of CRC patients and that it is a key gene driving CRC progression. Single nucleotide mutations in specific hotspot regions are able to change the conformation of the RAS active site, making it constitutively turned on (independently from ligand/receptor interaction) [4]. This phenomenon makes RAS-mutated CRC cells resistant to anti-EGFR monoclonal antibody-based treatments (cetuximab or panitumumab), so their use is recommended by the EMA (European Medical Agency) and FDA (Food and Drug Administration) only in mCRC patients with wild-type (wt) KRAS and NRAS. Notably, NRAS’ and KRAS’ most frequently altered codons are 12 and 13 in exon 2, 59–61 in exon 3, and 117 and 146 in exon 4 [5]. NRAS is mutated in fewer than 5% of mCRC patients [6,7].

In recent years, some studies have suggested a negative prognostic role for mutated KRAS (mKRAS) compared to wtKRAS in mCRC patients treated with standard first-line chemotherapies [8,9,10,11]. However, the prognostic value of specific KRAS mutations in fully tested RAS (“all RAS”) clinical series, including exons 2, 3, and 4, still remain unexplored and unclear. Many studies have been conducted predominantly reporting data on the association with CRC clinic-pathological characteristics or on methodological issues, in non-fully analysed RAS mutations [7]. Only one study has reported results on the prognostic role of differentiated KRAS mutations, pooling data from different “new generation” clinical trials, in which, however, different RAS testing methods were employed [12]. Interestingly, different RAS mutations (i.e., position and/or type of amino-acid substitutions in codons 12 and 13) associate with different levels of RAS-driven signals through modifications of the activation state of the protein itself and of the affinity with other downstream effectors [13]. Furthermore, it has been recently reported that specific mutations of KRAS are associated with chemoresistance in pancreatic cancer. In particular, pG12D mutants are more chemoresistant compared to wtKRAS, p.G12C, and p.G12V [14]. Altogether, these data suggest that oncogenic KRAS mutations are functionally different and could contribute to the biological and clinical heterogeneity of cancers. In particular, this functional diversity of mKRAS could translate to differential prognoses in CRC patients.

The present study was undertaken to describe both the incidence and prognostic role of specific KRAS mutations in a large cohort of mCRC patients analysed, treated, and followed-up at the same institution. The survival curves of mKRAS patients were also compared with those of a parallel internal cohort of wtKRAS patients.

## 2. Results

### 2.1. Clinico-Pathological Characteristics of Studied Cohort

Two hundred and six consecutive KRAS or NRAS mutant mCRC patients, treated at the Department of Abdominal Oncology of the National Cancer Institute of Naples from 2015 to 2019, were enrolled. Table 1 reports KRAS and NRAS specific mutations according to the clinical-pathological characteristics of the patients; statistical analyses (χ2 tests) were limited to 169 patients bearing p.G12D, p.G12V, p.G13D, p.G12A, p.G12C, and p.G12S KRAS mutations because of low numbers in the other variants. There were no significant differences among the most prevalent mutations (p.G12D, p.G12V, p.G13D, p.G12A, p.G12C, and p.G12S) in terms of age (<65 vs. >65 years), gender (male vs. female), grading (G1/G2 vs. G3), side of primary tumour (left vs. right), pT, and pN according to the American Joint Committee on Cancer (AJCC) 8th edition. Only two patients presented with mKRAS p.G13D and p.G12S and a concomitant BRAF mutation, p.V600E and p.D594N, respectively. 

### 2.2. Patients’ Treatments

Since all the patients were screened for RAS mutations to plan the treatment for advanced diseases (as institutional policy), Table 2 shows the metastatic involvement before starting the first-line chemotherapy, the type of first-line therapy, the best response, and the number of chemotherapy lines according to the indicated KRAS mutations in all the patients. No statistically significant imbalances were found between these characteristics for the most prevalent mutations. None of the KRAS-mutated patients received anti-EGFR-based treatments. The most applied first-line therapy was FOLFOX (Fluororuracil, Folinic Acid, and Oxaliplatin) or CAPOX (Capecitabine and Oxaliplatin) plus bevacizumab (73.9% of patients), while 17.2% received only chemotherapy because of specific medical conditions; the remaining 8.9% received FOLFIRI (Fluororuracil, Folinic Acid, and Irinotecan) plus aflibercept. More than half of the patients (56.8%) received a second-line treatment, based on FOLFIRI plus aflibercept in 76.1% of the cases. Thirty-six percent of the patients received more than two chemotherapy lines; in 83.6% of the patients, the third line consisted of the per os administration of regorafenib.

### 2.3. Overall Survival According to Specific KRAS Mutations

The time-to-outcome analysis was focused on p.G12D, p.G12V, p.G13D, p.G12A, p.G12C, and p.G12S KRAS mutations because of small events/patients in the other mutations’ classes. At a median follow-up of 25.6 months from the diagnosis of mCRC, there were 77 events (deaths) in KRAS-mutated patients. Table 3 shows the prognosis for each specific RAS mutation (median survivals with 95% CI or punctual survivals if <2 patients) and its incidence in our cohort. Interestingly, among the p.G12D, p.G12V, p.G13D, p.G12A, p.G12C, and p.G12S KRAS mutations, p.G12C and p.G12S were those with the worst prognoses (median survivals: 7.3 and 5.0 months, respectively; *p* = 0.0006 at Log Rank test). 

**Figure 1:**
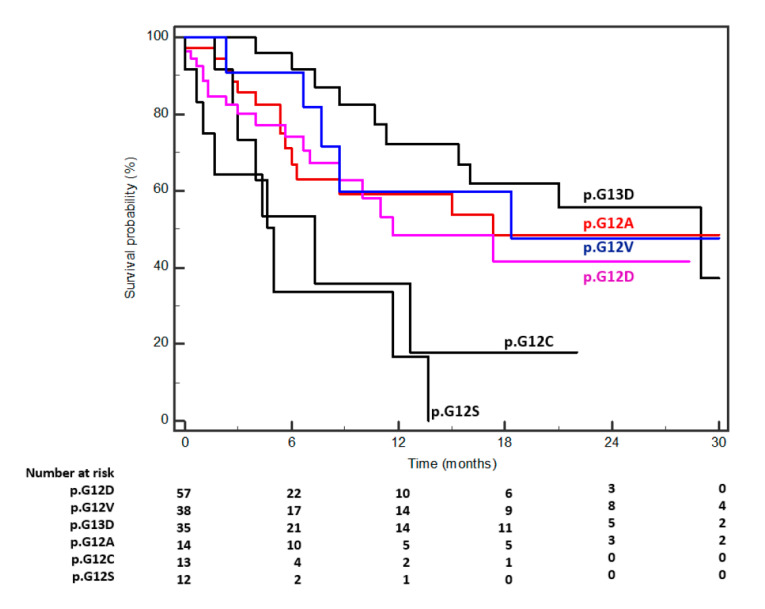
Kaplan–Meier survival curves according to different KRAS mutations (median survivals and *P* according to Log Rank tests are reported in Table 3).

Kaplan–Meier survival curves are depicted in Figure 1 and evidenced two prognostic clusters (Group B: p.G12D, p.G12V, p.G13D, and p.G12A, versus Group C: p.G12C and p.G12S) with an HR of 4.9 (CI: 2.1–11.5) for p.G12C/p.G12S and a median survival of 5.0 versus 18.3 months for group C and group B (*p* = 0.0002), respectively. 

Upon multivariate analysis (Table 4), the metastatic involvement (one site vs. multiple sites) and p.G12C/p.G12S KRAS mutations group (vs. other mutations) emerged as independent prognostic variables for survival. 

**Figure 2:**
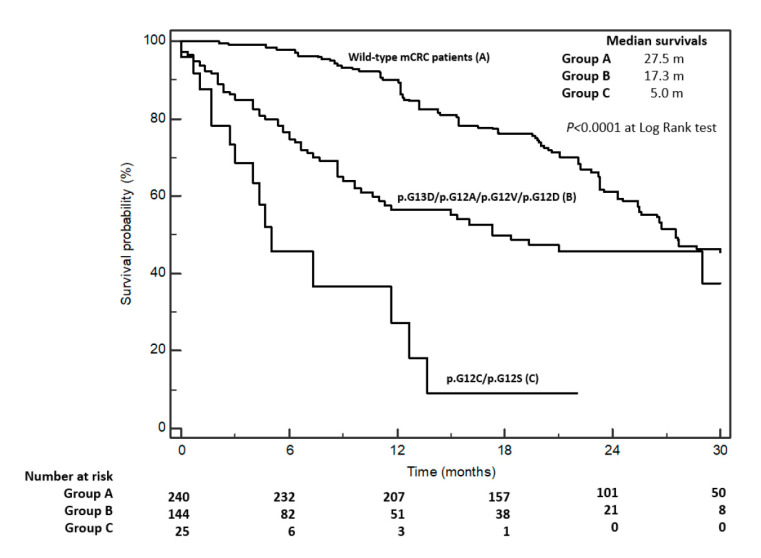
Kaplan–Meier survival curves according to three groups: A, wild-type KRAS metastatic colorectal cancer (mCRC) patients; B, pG13D/p.G12A/p.G12V/p.G12D mutated patients; C, pG12C/p.G12S mutated patients. Median survivals and *P* according to Log Rank tests are embedded into the figure area.

Although the present analyses focused on the detailed clinic-pathological and prognostic characteristics of RAS mutations, a parallel cohort of 240 wtRAS advanced patients, treated in the same period and selected applying the same criteria (see Methods), was analysed to provide a non-mutated control arm (group A). In this group, the prognosis was clearly biased by the application of anti-EGFR therapeutic strategies; however, it was useful to depict the general “prognostic differential” in a clinically homogeneous series of fully characterized wt “all RAS” patients. In Figure 2, we show and compare the prognosis of our wtRAS mCRC patients’ cohort (group A) with the homogeneous pooled groups of KRAS mutations (p.G13D/p.G12A/p.G12V/p.G12D (group B) and p.G12C/p.G12S (group C)). The median survivals were 27.5, 17.3, and 5.0 months for groups A, B, and C, respectively. The HR for C vs. B was 3.5 (CI: 1.12–12.33); for C vs. A, it was 5.96 (CI: 1.92–18.44). 

## 3. Discussion

The selection of patients in the era of targeted anti-cancer therapies is a crucial issue. The advent of molecular profiling in mCRC has made it possible to stratify patients and select the best therapies to be applied in clinical practice. To date, RAS mutation assessment is a solid hallmark for planning the therapeutic strategy in mCRC, since RAS-mutated patients do not benefit from anti-EGFR treatments [15]. Most molecularly oriented selection is based on KRAS evaluation, since the frequency of NRAS mutations accounts for less than 5% of RAS mutated CRC [6,7]. However, it is still not clear if different KRAS mutations associate with divergent outcomes. 

Modest et al. [12] reported from a pooled analysis of five randomized trials that, among specific KRAS mutations, the p.G12C variant predicted shorter survival (HR: 2.26, *p* = 0.001) according to multivariate analysis adjusted for sex, previous treatments, and burden of disease. However, this study suffered from some limitations: (i) the prognostic power of the comparison of the p.G12C variants against RAS wild-type tumours; (ii) the absence of complete RAS testing in at least three studies (FIRE-3, AIO KRK 0604, and RO91); (iii) the treatments’ heterogeneity (patients came from different institutions and not all the wild-type patients received anti-EGFR-based drugs); and (iv) the fact that RAS testing was performed using different techniques and, in some studies, in different laboratories rather than being centralized. Some of these limitations were identified and discussed by those authors who invoked the validation of their results through different study-sets. 

Notably, in the present report, progression-free survival was neither reported nor considered as a study objective because (i) the vital status is the most solid and reliable outcome for analysis and reporting in a retrospective analysis; (ii) in most cases, patients underwent different sequential treatments; and (iii) radiologic re-assessments were not homogeneous.

Interestingly, in our cohort, the negative impact of KRAS mutations was strong and clear for group C (p.G12C and p.G12S); for groups A and B, the curves tended to intersect, indicating that, in a later observation period, compensatory and dynamic phenomena made the prognosis similar. In other words, the clinical evolution of p.G12C and p.G12S mutations seems stably more negative. Liquid biopsy, whose dissertation is beyond the scope of this work, although still not a standard procedure, could represent a tool for monitoring tumour genetic dynamism. In fact, the liquid biopsies of the 12 patients included in group B showed the disappearance of KRAS mutations after third line therapies in five of them (manuscript in preparation). Conversely, in six group C patients that underwent liquid biopsies the KRAS mutations were confirmed. The analysis of these data is necessarily descriptive, being limited to hyper-selected patients. We have recently shown, in a very clean model of oligometastatic CRC, that the regressive trajectories of specific “backbone” mutations (such as in *SMAD4* and *KRAS*) can associate with a better clinical course [16]. In light of this, the hypothesis that group C KRAS mutations (p.G12C/p.G12S) might represent a more stable gain in the genetic dynamics of CRC is intriguing and deserves further specific studies.

Furthermore, in very recent years, the p.G12C mutation, quite frequent both in colorectal and in lung cancers [17], has been considered “druggable”; in fact, small molecules containing thiol reactive sites can covalently target the resulting cysteine and inhibit RAS functions [18,19]. The lack of cysteines in wtKRAS makes these compounds selective. Research is needed to develop and test these drugs in combination with chemotherapy in this poor prognosis CRC clinical setting. Interestingly, in a phase I study, AMG 510, a p.G12C inhibitor, showed a favourable safety profile, with no dose-limiting toxicities and no cumulative toxicities in the extended treatment of patients with pre-treated solid tumours harbouring that specific RAS mutation. Furthermore, a promising antitumor activity was observed in 34 and 36 patients affected by non-small cell lung cancer and CRC, respectively [20].

It cannot be ruled out that different specific mutations in KRAS can induce changes in the signal transduction pathways regulated by KRAS itself downstream. In fact, a shift in KRAS’ dynamics occurs in an allosteric manner, and a mutation can inflict changes in the protein’s dynamics in distant regions. Interestingly, some common oncogenic mutations, such as p.G12C, p.G12V, and p.Q61H, displayed weakened hypervariable region (HVR)–G domain association of KRAS [21]. Mutations in the GTP interaction site of the KRAS protein can influence the dynamics of the protein in distant regions (allosterically), influencing the interaction of the protein with other signal transduction interactors. For instance, the KRAS-mediated activation of PI3Kα occurs in an allosteric manner [22,23], and the G12 mutations stabilize the switch I region of RAS and tend to allosterically release the HVR from the catalytic domain, promoting RAS-PI3Kα recognition [24,25]. Interestingly, p.G12D, which is the most frequent KRAS mutant in cancer, appears to be most similar in its dynamics to wtKRAS, not at all altering the interaction with PI3Kα. 

Another important interaction of KRAS is with the downstream RAF kinase family proteins that, at the end, induce the stimulation of MAP kinase extracellular regulated kinases (ERKs). An additional finding of our work concerns the negative prognostic impact of pG12S mutations (collected into group C mutations). Interestingly, as occurs with pG12C, pG12S stabilizes the interactions with RAF (while a bulkier, hydrophobic p.G12V substitution leads to the destabilization of this interface) and increases its activation [26]. Therefore, it can be hypothesized that p.G12C and p.G12S KRAS mutants can induce more potent activation of RAF–ERK pathways than p.G12V and p.G12A (the latter is another substitution with a hydrophobic amino acid). In our series, patients harbouring RAF-over-interacting p.G12C and p.G12S KRAS mutations have a worse prognosis than those with RAF-hypo-interacting p.G12V and p.G12A and p.G12D KRAS mutations, which display dynamics more similar to wtKRAS. The patients harbouring the p.G13D KRAS mutation had better survival. In this light, it was reported that the kinetics of nucleotide exchange were essentially identical between all the G12 mutants and wtKRAS, with the exception of p.G13D, which has a GDP exchange rate 14 times faster than wtKRAS and a GTP exchange rate nine times faster than wtKRAS [27]. This faster exchange rate of GDP with GTP and vice versa makes p.G13D KRAS mutants independent from SOS (Son of Sevenless) exchange activity [28]. On the other hand, the duration of the activation of the downstream signalling components, including RAF, is likely attenuated. It was also observed that the GTP-binding pocket in the p.G12D mutant is more open than that of the wild-type and the p.G13D proteins, and the GAPs (Gtpase-Activating Proteins) activity of p.G13D is less affected than that of p.G12D, thus promoting the instability of GTP binding to the p.G13D mutant form [29]. These observations were confirmed in a clinical setting, as Tejpar et al. [30] demonstrated that the addition of cetuximab to first-line chemotherapy appears to benefit patients with p.G13D KRAS tumours, and the relative treatment effects were similar to those in patients with wtKRAS tumours but with lower absolute values. Similar results were obtained in two other independent trials [31,32]. Recently, it has been shown that mutations of KRAS are able to influence cancer metabolism; these mKRAS-induced modifications depend predominantly on tumor type. However, in general, mKRAS shifts the cancer metabolism towards anabolic pathways by regulating enzymes involved in glucose, amino acid, and fatty acid metabolism [33]. The modulation of these metabolic effects is emerging as a new therapeutic opportunity. Considering their differential prognostic impacts, future studies are needed to describe the metabolic adaptations induced by specific different KRAS mutations in CRC. This will allow the evaluation of any synergistic anti-cancer activity of mKRAS inhibition and cancer metabolism-oriented drugs. 

## 4. Materials and Methods 

STORIA (STudy of Ras mutatIons prognostic value in metAstatic colorectal cancer) is a retrospective analysis officially approved by the Scientific Directorate on 7 April 2020 (https://newportal.istitutotumori.na.it/comitati/comitato-etico/). The source of the data was the electronic database reporting the clinical records of mCRC patients enrolled at the Experimental Clinical Abdominal Oncology and SSD (*Struttura Semplice Dipartimentale*) Innovative Therapies for Abdominal Metastases of the Istituto Nazionale Tumori di Napoli, IRCCS “G. Pascale” from 2015 to 2019 and not amenable to the surgical removal of metastases. Oligometastatic patients, defined as those having 1–3 lesions per organ with a maximum of two involved organs, were excluded. To avoid negative prognostic interferences, the criteria for patients’ inclusion were established a priori and consisted of a Performance Status ECOG (Eastern Cooperative Oncology Group) of 0 or 1, age < 80 years, and life expectancy of at least three months. According to these criteria, 446 patients were selected: 206 had KRAS or NRAS mutations (see DNA sequencing), and 240, non-mutated RAS (wild-type RAS). All the patients received at least one chemotherapy line, and treatments were chosen at the oncologists’ discretion according to ESMO (European Society of Medical Oncology) guidelines. All the patients signed a written informed consent form before treatment administration and molecular pathology assessments. The primary outcome of this study was the analysis of the prognostic role of different KRAS mutations in terms of overall survival (OS). Patients harbouring exclusive BRAF mutations were not included in this analysis. 

### 4.1. Patient Management and Follow-Up

Total body CT (Computed Tomography) scans and CEA (CarcinoEmbryonic Antigen) monitoring were not centralized and were done every three months. The response to chemotherapy was evaluated according to RECIST (Response Evaluation Criteria in Solid Tumours v1.1). Complete response (CR) was defined as the complete disappearance of all detectable evidence of disease on total body computed tomography. Partial response (PR) was defined as at least a 30% decrease in the sum of the diameters of the target lesions. Stable disease (SD) was defined as everything between a 30% decrease and 20% growth in tumour size. Progressive disease (PD) was defined as at least a 20% increase in the sum of the diameters of the target lesions. Disease Control (DC) was the sum of CR + PR + SD.

### 4.2. DNA Extraction and Sequencing

The targeted sequencing of genomic DNA extracted from formalin-fixed paraffin-embedded (FFPE) tissues was performed as previously described [34]. Briefly, DNA was extracted from three 10 µm FFPE sections using the QIAamp DNA FFPE Tissue kit (Qiagen, Hilden, Germany) and the QIAcube apparatus (Qiagen). The DNA quantity was evaluated with the dsDNA HS assay kit using the Qubit 2.0 Fluorometer (Invitrogen, Monza, Italy). Tumour samples were sequenced with the Oncomine Solid Tumour DNA kit (Thermo Fisher Scientific, Waltham, MA, USA) covering the hotspot variants and actionable mutations of 22 genes involved in colon cancer. Ten nanograms of genomic DNA (gDNA) was used to prepare libraries according to the manufacturer’s instructions. The amplified libraries were sequenced on the Ion Torrent PGM semiconductor (https://www.thermofisher.com/it/en/home/life-science/sequencing/next-generation-sequencing/ion-torrent-next-generation-sequencing-workflow.html), the data were analysed using the Torrent Suite Software v5.0 (Thermo Fisher Scientific), and the obtained variants were confirmed using the integrative genome viewer (IGV) from the Broad Institute. The limit of mutation detection (LOD) of the tissue NGS approach is 2% allelic frequency. The reference sequences were NM_004958.4 for KRAS and NM_002524.4 for NRAS. The mutations were also reported according to ClinVar identifier numbers (https://www.ncbi.nlm.nih.gov/clinvar/intro/).

### 4.3. Statistical Analyses, Study Design, and Data Presentation

Associations between KRAS mutations and clinical and pathologic variables were evaluated by the χ2 test. *p* < 0.05 was considered statistically significant. The primary outcome measure was the OS, measured from the start of the first-line chemotherapy until death from any cause. The Kaplan–Meier product limit method was applied to graph OS. A pre-specified hypothesis based on the solid assumption that KRAS mutations have a negative prognostic impact in mCRC was used to size the study. For this reason, the STORIA study aimed to recruit at least 110 patients per group (mutated versus non-mutated, powered at 80% to detect a Hazard Ratio—HR—of 0.65, considering a minimal difference in median survival among poor and good prognosis groups at 6 months). Univariate analysis was performed with the Log-Rank test. Multivariate analysis was performed through Cox proportional-hazards regression in order to analyse the effect of several risk factors (co-variates) on OS. The HR is the estimate of the end-point probability, and it can be interpreted as the instantaneous relative risk of an event (death), at any time, for an individual with the risk factor present compared with an individual with the risk factor absent, given both individuals are the same according to all other covariates. Covariates were selected based upon a consensus after discussion between the authors and were dichotomized: age < 65 vs. age ≥ 65, male vs. female, left sided vs. right sided, one involved organ vs. two or more, response to first-line chemotherapy, and specific KRAS mutations vs. other KRAS mutations. The 95% confidence intervals (CI) of the HRs are also reported. Statistical analysis was performed using the MedCalc^®^ 9.3.7.0 (www.medcalc.org) and Excel software.

## 5. Conclusions

Our data provide direct evidence that CRC driven by pG12C/p.G12S mKRAS is a more aggressive clinical subtype and is the ideal setting in which to test potentiated and/or innovative therapeutic strategies including small inhibitors of mutated KRAS (both in monotherapy or in association with standard chemotherapy). Additionally, on the basis of these results, we are prospectively following stage I to III mKRAS CRC patients after radical surgery to observe any difference in the recurrence rate justifying the proposal of a more intensive risk-adapted adjuvant approach in specific mKRAS patients’ subsets.

## Figures and Tables

**Table 1:** Clinico-pathological Characteristics Sccording to RAS Mutations.

Gene	Mutation (Proteic Change)	Age			Gender			Grading			Side of Primary Tumor			pT *				Lymph Node Involvement (pN) *			
		<65	≥65	*p*	M	F	*p*	G1/G2	G3	*p*	Left	Right	*p*	T1/T2	T3	T4	*p*	0	1–3	>3	*p*
**KRAS**																					
	p.G12D	22	35		27	30		10	47		26	31		12	30	10		1	6	45	
	p.G12V	17	21		20	18		6	32		14	24		11	15	9		1	9	25	
	p.G13D	15	20		18	17		4	31		12	23		7	16	6		0	5	24	
	p.G12A	6	8		11	3		4	10		4	10		2	5	4		1	3	7	
	p.G12C	5	8		5	8		3	10		2	11		4	5	3		0	2	10	
	p.G12S	5	7	0.99	4	8	0.22	2	10	0.78	3	9	0.33	2	5	3	0.94	0	1	9	0.59
	p.A146T	2	6		5	3		4	4		4	4		1	2	3		2	1	3	
	p.A146V	1	2		2	1		0	3		1	2		1	1	0		1	0	1	
	p.K117N	2	1		0	3		0	3		0	3		0	1	2		0	1	2	
	p.G13C	0	3		3	0		1	2		1	2		0	1	1		0	0	2	
	p.G13R	1	1		0	2		1	1		1	1		2	0	0		1	0	1	
	p.G12_G13insG	0	1		1	0		0	1		0	1		0	1	0		0	0	1	
	p.G12F	0	1		1	0		0	1		1	0		0	1	0		0	0	1	
	p.G13D; pG12D	1	0		1	0		0	1		1	0		1	0	0		0	0	1	
	p.A59E	0	1		1	0		0	1		0	1		0	1	0		1	0	0	
**NRAS**																					
	p.G12C	2	2		3	1		1	3		1	3		0	3	0		0	1	2	
	p.Q61H	1	3		2	2		1	3		0	4		0	3	0		0	0	3	
	p.G12R	1	1		2	0		0	2		0	2		1	0	1		0	0	2	
	p.Q61E	0	1		0	1		0	1		1	0		0	1	0		0	0	1	
	p.Q61L	0	1		1	0		0	1		0	1		0	1	0		1	0	0	
	p.Q61R	0	1		0	1		1	0		0	0		0	1	0		0	0	1	
	p.V14I	1	0		0	1		0	1		1	1		0	0	1		0	0	1	

pT: pathological staging of primary tumor according to AJCC. pN: pathological staging of loco-regional lymph node involvement according to AJCC. * The row sum does not correspond to the total number of patients because some of them (34) did not receive surgical removal of primary tumor. Statistical analysis was applied to cases above the discontinuous line.

**Table 2:** Tumor burden, Response to First-line Chemotherapy, and time-on-therapy According to RAS Mutations.

Gene	Mutation (Proteic Change)	Metastatic Involvement				Type of First-Line CT			Best Response to First-Line CT				No. of Chemotherapy Lines				Time on Therapy (Months) *
		One Site	Two or More Sites	Multiple Sites Including Peritoneum	*p*	CT	CT/AA	*p*	CR, PR or SD	PD	NA	*p*	1	2	>2	*p*	Median (Range)
**KRAS**																	
	p.G12D	12	33	12		9	48		27	22	8		57	31	20		19.2 (11.7–23.7)
	p.G12V	11	18	9		5	33		19	12	7		38	18	13		18.8 (8.5–23.2)
	p.G13D	7	22	6		6	29		19	11	5		35	20	13		19.1 (8.8–25.0)
	p.G12A	2	8	4		3	11		7	7	0		14	9	7		18.2 (11.1–23.6)
	p.G12C	5	5	3		2	11		4	9	0		13	6	2		10.7 (4.3–23.1)
	p.G12S	2	7	3	0.87	4	8	0.71	2	8	2	0.14	12	4	0	0.76	5.9 (3.9–13.1)
	p.A146T	3	2	3		2	6		5	3	0		8	6	6		16.9 (13.3–21.8)
	p.A146V	2	1	0		2	1		2	0	1		3	3	2		16.3, 18.6, 18.9
	p.K117N	0	1	2		0	3		1	2	0		3	2	2		17.4, 19.0, 19.2
	p.G13C	1	1	1		1	2		2	1	0		3	3	2		17.2, 21.6, 22.4
	p.G13R	2	0	0		0	2		2	0	0		2	2	0		10.4, 19.5
	p.G12_G13insG	0	1	0		0	1		1	0	0		1	1	1		12.2
	p.G12F	0	1	0		0	1		0	1	0		1	1	1		20.6
	p.G13D; pG12D	1	0	0		1	0		0	0	1		1	1	0		22.7
	p.A59E	0	1	0		1	0		0	1	0		1	1	1		25.2
**NRAS**																	
	p.G12C	0	4	0		1	3		3	1	0		4	2	0		15.5 (6.6–18.0)
	p.Q61H	0	4	0		0	4		1	1	2		4	3	1		11.6 (8.4–19.3)
	p.G12R	1	0	1		1	1		2	0	0		2	0	0		15.3, 19.2
	p.Q61E	0	1	0		0	1		1	0	0		1	1	1		11.4
	p.Q61L	0	1	0		0	1		0	1	1		1	1	0		23.2
	p.Q61R	0	1	0		0	1		0	0	1		1	1	0		21.1
	p.V14I	0	0	1		1	0		1	0	0		1	1	1		18.7

AA: Anti-Angiogenic drug; CR: Complete Response; CT: ChemoTherapy; NA: Not Assessable; PD: Progressive Disease; PR: Partial Response. SD: Stable Disease. Statistical analysis was applied to cases above the discontinuous line. * Cumulative time spent on therapy (also including “maintenance therapy”).

**Table 3:** Univariate Analysis of RAS Mutations’ Prognostic Power.

Gene	Proteic Change	No. of Events/Patients	Mutation Incidence (%)	ClinVar ID	Median Survival (Months)	95% CI	*p* at Log Rank Test
**KRAS**							
	p.G12D	19/57	27.7	27261	11.6	8.6–17.3	
	p.G12V	15/38	18.4	27622	17.3	6.0–31.6	
	p.G13D	10/35	17.0	27619	27.0	15.3–29.0	
	p.G12A	5/14	6.8	54289	16.3	7.6–18.3	
	p.G12C	7/13	6.3	27617	7.3	1.6–12.6	
	p.G12S	8/12	5.8	12584	5.0	3.0–11.6	*p* = 0.0006
	p.A146T	6/8	3.9	194404	19.3	6.4–21.4	
	p.A146V	2/3	1.4	362841	2.0	0.6–2.0	
	p.K117N	2/3	1.4	362843	4.6	2.3–6.1	
	p.G13C	1/3	1.4	54290	29.8	5.4–36.3	
	p.G13R	1/2	0.9	27632	0.6 and 6.3	NA	
	p.G12_G13insG	0/1	0.5	NR	19.6	NA	
	p.G12F	1/1	0.5	NR	1.4	NA	
	p.G13D; pG12D	0/1	0.5	NR	19.6	NA	
	p.A59E	0/1	0.5	NR	10.0	NA	
**NRAS**							
	p.G12C	3/4	1.9	48938	4.8	2.9–7.9	
	p.Q61H	2/4	1.9	359197	9.0	8.6–9.9	
	p.G12R	1/2	0.9	48939	1.6 and 4.6	NA	
	p.Q61E	1/1	0.5	362754	6.2	NA	
	p.Q61L	0/1	0.5	362753	20.3	NA	
	p.Q61R	1/1	0.5	28939	5.5	NA	
	p.V14I	1/1	0.5	27628	9.6	NA	

CI: Confidence Interval; NA: Not Assessable; NR: Not Reported. Statistical analysis was applied to cases above the discontinuous line.

**Table 4:** Multivariate Analysis of RAS Mutations’ Prognostic Power.

Co-Variate	Dichotomization	Median Survivals	No. of Events/Patients	*p* at Univariate	HR	95% CI	*p* at Multivariate
**Age**	<65 year vs. ≥65 year	17.3 vs. 12.6	40/82 vs. 46/124	0.4088	0.82	0.52–1.29	0.548
**Gender**	M vs. F	13.6 vs. 15.0	35/107 vs. 51/99	0.8902	0.96	0.62–1.50	0.872
**Side**	L vs. R	15.3 vs. 13.6	32/73 vs. 54/133	0.6084	0.88	0.56–1.39	0.309
**Metastatic involvement**	1 site vs. >1	NR vs. 9.6	12/49 vs. 74/157	<0.0001	0.36	0.22–0.58	0.001
**Response to first-line CT**	DC vs. not DC	29.0 vs. 9.0	37/98 vs. 30/80	0.0032	0.46	0.28–0.77	0.073
KRAS mutations *	p.G12D vs. other mut	11.6 vs. 15.3	19/57 vs. 45/112	0.6510	0.87	0.50–1.53	0.669
	p.G12V vs. other mut	17.3 vs. 13.6	15/38 vs. 49/131	0.5684	0.84	0.48–1.48	0.580
	p.G13D vs. other mut	27.0 vs. 11.6	10/35 vs. 54/134	0.0384	0.55	0.31–0.96	0.165
	p.G12A vs. other mut	16.3 vs. 15.0	5/14 vs. 59/155	0.4104	0.71	0.32–1.58	0.634
	p.G12C/p.G12S vs. other mut	5.0 vs. 18.3	15/25 vs. 49/144	0.0002	4.99	2.15–11.5	0.002

* The analysis is limited to KRAS p.G12D, p.G12V, p.G13D, p.G12A, p.G2C, and p.G12S patients. CI: Confidence Interval; DC: Disease Control; F: Female; HR: Hazard Ratio; L: Left; M: Male; mut: KRAS mutations; NR: Not Reached; R: Right.

## References

[1] Ferlay J., Colombet M., Soerjomataram I., Mathers C., Parkin D.M., Piñeros M., Znaor A., Bray F. (2019). Estimating the global cancer incidence and mortality in 2018: GLOBOCAN sources and methods. Int. J. Cancer.

[2] Vatandoust S., Price T.J., Karapetis C.S. (2015). Colorectal cancer: Metastases to a single organ. World J. Gastroenterol..

[3] Dekker E., Tanis P.J., Vleugels J.L.A., Kasi P.M., Wallace M.B. (2019). Colorectal cancer. Lancet.

[4] Simanshu D.K., Nissley D.V., McCormick F. (2017). RAS proteins and their regulators in human disease. Cell.

[5] Normanno N., Tejpar S., Morgillo F., De Luca A., Van Cutsem E., Ciardiello F. (2009). Implications for KRAS status and EGFR-targeted therapies in metastatic CRC. Nat. Rev. Clin. Oncol..

[6] Jinesh G.G., Sambandam V., Vijayaraghavan S., Balaji K., Mukherjee S. (2018). Molecular genetics and cellular events of K-Ras-driven Tumorigenesis. Oncogene.

[7] Waring P., Tie J., Maru D., Karapetis C.S. (2016). RAS Mutations as Predictive Biomarkers in Clinical Management of Metastatic Colorectal Cancer. Clin. Colorectal Cancer.

[8] Petrelli F., Coinu A., Cabiddu M., Ghilardi M., Barni S. (2013). KRAS as Prognostic Biomarker in Metastatic Colorectal Cancer Patients Treated with Bevacizumab: A Pooled Analysis of 12 Published Trials. Med. Oncol..

[9] Smith J.C., Brooks L., Hoff P.M., McWalter G., Dearden S., Morgan S.R., Wilson D., Robertson J.D., Jürgensmeier J.M. (2013). KRAS Mutations Are Associated with Inferior Clinical Outcome in Patients with Metastatic Colorectal Cancer, but Are Not Predictive for Benefit with Cediranib. Eur. J. Cancer.

[10] Koike J., Ushigome M., Funahashi K., Shiokawa H., Kaneko T., Arai K., Matsuda S., Kagami S., Suzuki T., Kurihara A. (2014). Significance of KRAS Mutation in Patients Receiving mFOLFOX6 with or without Bevacizumab for Metastatic Colorectal Cancer. Hepatogastroenterology.

[11] Petrelli F., Coinu A., Cabiddu M., Borgonovo K., Lonati V., Ghilardi M., Barni S. (2015). Prognostic factors for survival with bevacizumab-based therapy in colorectal cancer patients: A systematic review and pooled analysis of 11,585 patients. Med. Oncol..

[12] Modest D.P., Ricard I., Heinemann V., Hegewisch-Becker S., Schmiegel W., Porschen R., Stintzing S., Graeven U., Arnold D., von Weikersthal L.F. (2016). Outcome according to KRAS-, NRAS- and BRAF-mutation as well as KRAS mutation variants: Pooled analysis of five randomized trials in metastatic colorectal cancer by the AIO colorectal cancer study group. Ann Oncol..

[13] Li S., Balmain A., Counter C.M. (2018). A model for RAS mutation patterns in cancers: Finding the sweet spot. Nat. Rev. Cancer.

[14] Mukhopadhyay S., Goswami D., Adiseshaiah P.P., Burgan W., Yi M., Guerin T.M., Kozlov S.V., Nissley D.V., McCormick F. (2020). Undermining Glutaminolysis Bolsters Chemotherapy while NRF2 Promotes Chemoresistance in KRAS-Driven Pancreatic Cancers. Cancer Res..

[15] Van Cutsem E., Cervantes A., Adam R., Sobrero A., Van Krieken J.H., Aderka D., Aranda Aguilar E., Bardelli A., Benson A., Bodoky G. (2016). ESMO consensus guidelines for the management of patients with metastatic colorectal cancer. Ann. Oncol..

[16] Ottaiano A., Circelli L., Lombardi A., Scala S., Martucci N., Galon J., Buonanno M., Scognamiglio G., Botti G., Hermitte F. (2020). Genetic trajectory and immune microenvironment of lung-specific oligometastatic colorectal cancer. Cell Death Dis..

[17] Visscher M., Arkin M.R., Dansen T.B. (2016). Covalent targeting of acquired cysteines in cancer. Curr. Opin. Chem. Biol..

[18] Janes M.R., Zhang J., Li L.S., Hansen R., Peters U., Guo X., Chen Y., Babbar A., Firdaus S.J., Darjania L. (2018). Targeting KRAS mutant cancers with a covalent G12C-specific inhibitor. Cell.

[19] Patricelli M.P., Janes M.R., Li L.S., Hansen R., Peters U., Kessler L.V., Chen Y., Kucharski J.M., Feng J., Ely T. (2016). Selective inhibition of oncogenic KRAS output with small molecules targeting the inactive state. Cancer Discov..

[20] Govindan R., Fakih M.G., Price T.J., Falchook G.S., Desai J., Kuo J.C., Strickler J.H., Krauss J.C., Li B.T., Denlinger C.S. (2019). Phase 1 study of AMG 510, a novel molecule targeting KRAS G12C mutant solid tumors. Ann. Oncol..

[21] Pantsar T. (2019). The current understanding of KRAS protein structure and dynamics. Comput. Struct. Biotechnol. J..

[22] Zhang M., Jang H., Nussinov R. (2019). The structural basis for Ras activation of PI3Kα lipid kinase. Phys. Chem. Chem. Phys..

[23] Zhang M., Jang H., Nussinov R. (2019). The mechanism of PI3Kα activation at the atomic level. Chem. Sci..

[24] Lu S., Jang H., Muratcioglu S., Gursoy A., Keskin O., Nussinov R., Zhang J. (2016). Ras conformational ensembles, allostery, and signalling. Chem. Rev..

[25] Lu S., Jang H., Gu S., Zhang J., Nussinov R. (2016). Nussinov Drugging Ras GTPase: A comprehensive mechanistic and signaling structural view. Chem. Soc. Rev..

[26] Mehaffey M.R., Schardon C.L., Novelli E.T., Cammarata M.B., Webb L.J., Fast W., Brodbelt J.S. (2019). Investigation of GTP-dependent dimerization of G12X K-Ras variants using ultraviolet photodissociation mass spectrometry. Chem. Sci..

[27] Misale S., Fatherree J.P., Cortez E., Li C., Bilton S., Timonina D., Myers D.T., Lee D., Gomez-Caraballo M., Greenberg M. (2019). KRAS G12C NSCLC Models Are Sensitive to Direct Targeting of KRAS in Combination with PI3K Inhibition. Clin. Cancer Res..

[28] Hunter J.C., Manandhar A., Carrasco M.A., Gurbani D., Gondi S., Westover K.D. (2015). Biochemical and Structural Analysis of Common Cancer-Associated KRAS Mutations. Mol. Cancer Res..

[29] Chen C.C., Er T.K., Liu Y.Y., Hwang J.K., Barrio M.J., Rodrigo M., Garcia-Toro E., Herreros-Villanueva M. (2013). Computational analysis of KRAS mutations: Implications for different effects on the KRAS p.G12D and p.G13D mutations. PLoS ONE.

[30] Tejpar S., Celik I., Schlichting M., Sartorius U., Bokemeyer C., Van Cutsem E. (2012). Association of KRAS G13D tumor mutations with outcome in patients with metastatic colorectal cancer treated with first-line chemotherapy with or without cetuximab. J. Clin. Oncol..

[31] Messner I., Cadeddu G., Huckenbeck W., Knowles H.J., Gabbert H.E., Baldus S.E., Schaefer K.L. (2013). KRAS p.G13D mutations are associated with sensitivity to anti-EGFR antibody treatment in colorectal cancer cell lines. J. Cancer Res. Clin. Oncol..

[32] De Roock W., Jonker D.J., Di Nicolantonio F., Sartore-Bianchi A., Tu D., Siena S., Lamba S., Arena S., Frattini M., Piessevaux H. (2010). Association of KRAS p.G13D mutation with outcome in patients with chemotherapy-refractory metastatic colorectal cancer treated with cetuximab. JAMA.

[33] Pupo E., Avanzato D., Middonti E., Bussolino F., Lanzetti L. (2019). KRAS-Driven Metabolic Rewiring Reveals Novel Actionable Targets in Cancer. Front. Oncol..

[34] Normanno N., Rachiglio A.M., Lambiase M., Martinelli E., Fenizia F., Esposito C., Roma C., Troiani T., Rizzi D., Tatangelo F. (2015). Heterogeneity of KRAS, NRAS, BRAF and PIK3CA mutations in metastatic colorectal cancer and potential effects on therapy in the CAPRI GOIM trial. Ann. Oncol..

